# Acupoint injection versus sacral canal injection in lumbar disc herniation: A protocol of randomized controlled trial

**DOI:** 10.1097/MD.0000000000023000

**Published:** 2020-11-13

**Authors:** Wei Li, Huaying Wang, Lijun Wang, Peng Tang, Yaokai Huang

**Affiliations:** The People's Hospital of Dazu District, Dazu, Chongqing, China.

**Keywords:** acupoint injection, lumbar disc herniation, protocol, randomized controlled trial, sacral canal injection

## Abstract

**Background:**

Both acupoint injection and sacral canal injection are widely adopted in the treatment of lumbar disc herniation (LDH), but there are still doubts about the effectiveness and safety of the 2 methods. Therefore, the objective of the randomized controlled trial is to evaluate the effectiveness and safety of acupoint injection and sacral canal injection in the treatment of LDH.

**Method:**

This is a prospective randomized controlled trial to study the effectiveness and safety of acupoint injection and sacral canal injection in the treatment of LDH. With the approval by the clinical research ethics committee of our hospital, patients were randomly included into 1 of 2 treatment protocols:

(1) acupoint injection group and
(2) sacral canal injection group.

Patients, doctors, nurses, and research assistants responsible for collecting data were blinded to group allocation. Main outcome observation indicator: visual analogue scale; secondary outcome observation indicator: Oswestry disability index scores; paresthesia score; adverse reactions. Data were analyzed using the statistical software package SPSS version 25.0 (Chicago, IL).

**Discussion:**

The effectiveness and safety of acupoint injection and sacral canal injection in the treatment of LDH were evaluated in this study, and the results of this trial would establish clinical evidence for the adoption of acupoint injection or sacral canal injection to treat LDH.

**Trial registration number:**

DOI 10.17605 / OSF.IO / VTFUD

## 1 Introduction

Lumbar disc herniation (LDH) may have a series of symptoms caused by displacement of nucleus pulposus between discs and compression of peripheral nerve roots. Its clinical signs include radicular symptoms, paresthesia, and weakness of lumbosacral nerve distribution area.^[1]^ Non-surgical treatment is preferred for patients with LDH.^[2]^ Many conservative treatments are also available, including oral and external drugs, physical therapy, spinal manipulation, traction, epidural steroid injections, transcutaneous electrical stimulation, acupuncture, etc.^[3]^ Acupoint injection is based on traditional acupuncture theory, injecting drugs into specific acupoints, and achieving the purpose of treatment by stimulating the acupoints.^[4]^ Acupoint injection and meridian acupoint stimulation have a synergistic effect, and this method has a better effect than traditional acupuncture or simple intramuscular injection.^[5]^ It has already been applied to the treatment of myofasciitis, knee osteoarthritis, external humeral epicondylitis, and other muscle and joint diseases.^[6–8]^ Acupoint injection has been clinically proven to relieve the pain of patients with LDH and improve their ability of daily living.^[9,10]^ Sacral canal injection is another commonly used injection therapy for the treatment of chronic low back pain.^[11]^ The drug is injected into the epidural space from hiatus sacralis through a needle to achieve anti-inflammatory, analgesic, and neurotrophic effects.

At present, both injection methods have been widely adopted in the treatment of LDH, but there is a lack of comparative studies on the effectiveness and safety of the 2. Therefore, the objective of the randomized controlled trial is to evaluate the effectiveness and safety of acupoint injection and sacral canal injection in the treatment of LDH.

## 2 Materials and methods

### 2.1 Study design

**Figure 1:**
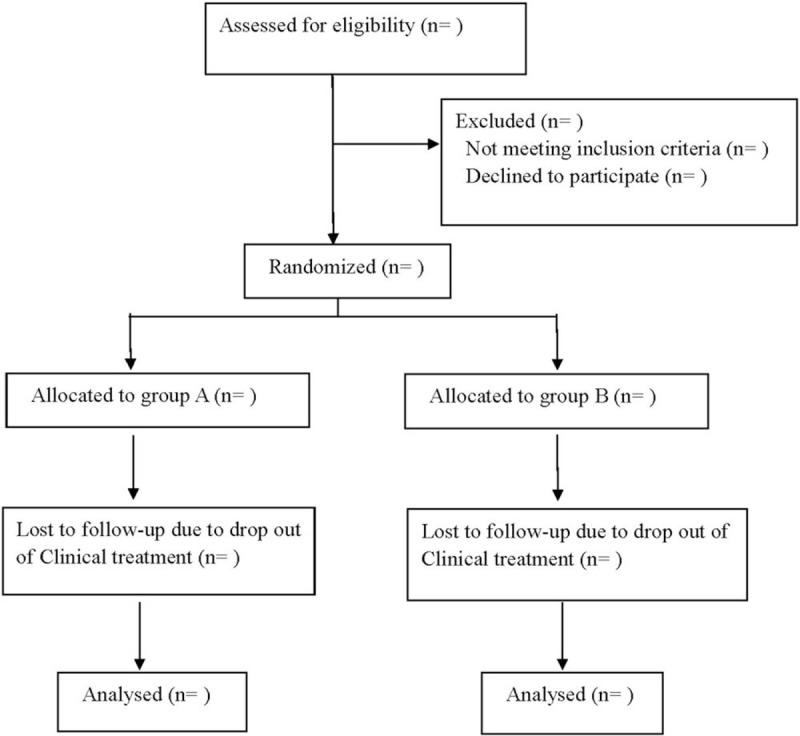
Flow diagram of the study.

This is a prospective randomized controlled trial to study the safety and effectiveness of acupoint injection and sacral canal injection in the treatment of LDH. We followed the Consolidated Standards of Reporting Trials guidelines for reporting randomized trials and provided a Consolidated Standards of Reporting Trials flow diagram (Fig. 1) and the Standard Protocol Items: Recommendations for Interventional Trials 2013 statement.

### 2.2 Ethics and registration

This research protocol complied with the Declaration of Helsinki and was approved by the clinical research ethics committee of our hospital. This trial has already been registered in open science framework. (Registration number: DOI 10.17605 / OSF.IO / VTFUD). Before random allocation, all patients needed to sign a written informed consent that they were free to choose whether to continue the trial at any time.

### 2.3 Sample size

The calculation of sample size is based on the main results (visual analogue scale [VAS]), According to the results of the pilot study, it is estimated that the average score of the Acupoint injection group was 3.5, and the standard deviation was 0.65. The average of the Sacral canal injection group was 4.03 and the standard deviation was 1.12. formula for calculating the sample size is as follows

$$n=n1=n2\frac{{\left(u_{\alpha }+u_{\beta }\right)}^{2}\times \sigma^{2}}{\delta^{2}}\times 2$$

At the 5% significance level, a total of 51 patients are needed in each group to achieve 80% power. The estimated dropout rate is 20%, and a total of 64 patients have been included in each group.

### 2.4 Patients

Inclusion criteria: Patients were diagnosed with LDH after CT or MRI; no indications for surgery, suitable for conservative treatment; 19 to 65 years old; VAS greater than 40 points.

Exclusion criteria: ① existence of skin disease or ulceration in the treatment area; ② existence of spinal trauma or surgery history; ③ existence of coagulation dysfunction; ④ lumbar fracture, tumor, or infection; ⑤ existence of mental disease under treatment, such as depression, schizophrenia, etc; ⑥ received oral drugs or other physical therapy in the past week; ⑦ allergic to injection drugs; ⑧ unable to understand the research protocol after explanation or unwilling to participate.

### 2.5 Randomization and blinding

Patients were randomly divided into 1 of 2 treatment protocols:

(A) acupoint injection group,
(B) sacral canal injection group.

Randomization was performed without any stratification. Randomization listings were prepared with a probability of 1:1 and after that, randomization letters were printed according to the results of the randomization. After the patient had given consent, a member of the in-hospital clinical study center chose 1 of the 2 letters and the patient was assigned to 1 group. Patients, doctors, nurses, and research assistants collecting data were blinded to group allocation.

### 2.6 Interventions

(1) Acupoint injection: the patient took the prone position to expose the skin of waist and buttock and popliteal space. Bilateral Shenshu acupoints, Ciliao acupoints, Yaoyangguan acupoints, and Weizhong acupoint on the affected side were located. After local disinfection, No. 7 needle was connected with 10 mL syringe, and was stabbed the acupoints vertically and slowly. After the patient had soreness and swelling and there was no blood return after aspiration, drugs were injected. Each acupoint was injected with about 0.5 to 1 mL, and the depth of acupuncture was about 2 to 3 cm. Drug composition: 500ug vitamin B12 was diluted with normal saline to 10 mL. After the injection, the patient was prone to rest for 30 minutes to observe whether there was any discomfort.
(2) Sacral canal injection: the patient took the prone position, a soft pillow was placed under the pelvis, and a depression was palpable about 1 cm from the top of coccyx, that is, hiatus sacralis. After local disinfection, 2% lidocaine was used for local anesthesia, and then a 20 mL syringe was connected with No. 7 syringe needle and sacral canal stabbing was performed at 30° to 45°. After the feeling of “failure” and there was no blood or cerebrospinal fluid outflow, drugs were injected. The injection should have no obvious resistance, 20 mL each time. Drug composition: 2 mL 2% lidocaine, 5 to 10 mL triamcinolone acetonide, 500ug vitamin B12 that was diluted with normal saline to 20 mL. After the injection, the patient was prone to rest for 30 minutes to observe whether there was any discomfort.

All patients received injections once a week for a total of 4 weeks, and all injections were performed by the same surgeon.

### 2.7 Outcome measures

Secondary outcomes: ① Oswestry disability index scores, which would include 10 categories: pain intensity, personal care, lifting, sitting, walking, standing, sleeping, social life, travel, and degree of pain. The higher the score, the more severe the disease. The Oswestry disability index was a reliable and valid scale suitable for measurement of disability in patients with low back pain^[14]^; ② paresthesia (such as numbness, tingling), as the secondary common symptom of sciatica,^[15]^ paresthesia would also be used to evaluate the recovery period. We also used VAS evaluation, where “0” meant no numbness and tingling, “100” meant unbearable numbness and tingling. ③ Adverse reactions, we would count the number of adverse reactions, including fainting during acupuncture, allergic reactions, worsening conditions, etc.

### 2.8 Data collection and management

In order to evaluate the treatment effect, data would be collected at baseline. We would collect data before treatment, after each treatment, and the 7^th^, 14^th^, 28^th^ days after the end of treatment based on the outcome indicators. All data would be collected by a single assistant. All data would be stored and kept separately, and the access to the database would be restricted to the researchers in this study team.

### 2.9 Statistical analysis

Data were analyzed using the statistical software package SPSS version 25.0 (Chicago, IL). Continuous variables were described as the mean ± standard deviation, and differences between groups were analyzed using a series of one-way analysis of variance with Bonferroni's post-hoc test, while differences between groups over time were analyzed using multi-way analysis of variance with Bonferroni post-hoc test. Categorical variables were described as the number (%), and were analyzed by Fisher exact test. A *P* value of < .05 was considered statistically significant.

## 3 Discussion

LDH can cause physical deterioration, long-term low back and leg pain, neurologic impairment, and long-term socio-economic problems caused by treatment-related costs,^[16]^ most patients can be treated conservatively. Sacral canal injection treatment is also called sacral canal epidural drug injection treatment. The drug is injected into the epidural space through sacral canal to directly act on the nerve roots and spinal cord at the lesion, block pain transmission and vicious circle, promote local inflammatory absorption, so as to achieve the purpose of relieving pain and improving function, which has already been confirmed to have a definite effect in the treatment of low back pain with nerve root radiating pain.^[17]^ Animal trials have found that after sacral canal injection of steroid drugs, the content of some inflammatory mediators PGE2, IL-1, and IL-6 in the nerve root compression area significantly decreases, which can inhibit local inflammatory response and reduce nerve root sensitivity.^[18]^ Acupoint injection is based on the traditional theories of traditional Chinese medicine. LDH belongs to the categories of “back pain” and “arthralgia” in traditional Chinese medicine.^[19]^ Due to the invasion of wind-cold-wetness evil, injuries from falls, fractures, contusions and strains and chronic strain, meridians are blocked, and the blood flow is not smooth with symptoms such as pain, numbness, and inhibited bending and stretching.^[20]^ Shenshu acupoint, Ciliao acupoint, Yaoyangguan acupoint, and Weizhong acupoint selected for acupoint injection can invigorate yang qi, relax muscles and collaterals, relieve pain, promote blood circulation to remove blood stasis and improve lower limb numbness. Modern studies have found that Shenshu acupoint can increase the speed of sensory and motor nerve transmission in the lower limbs,^[21]^ and stimulating Weizhong acupoint can promote blood circulation, inhibit the release of inflammatory factors such as SP, IL-6, PGE2, and improve pain.^[22]^

At present, 2 injection treatments have been adopted alone or in combination in the treatment of LDH,^[23,24]^ but there is a lack of comparative studies on the effectiveness and safety of the 2. The results of this study will suggest clinical evidence comparing acupoint injection with sacral canal injection by providing data about the changes in various measurements from the rigorously conducted. This study will provide the basis for clinicians to choose acupoint injection or sacral canal injection to treat LDH.

The following limitations may also be found in this study: due to the intervention method, the operator and the patient cannot be strictly double-blind, and there may be a certain bias; factors such as age and course of patients included in this study may have a certain impact on the results.

## Author contributions

**Conceptualization:** Wei Li.

**Data curation:** Lijun Wang.

**Formal analysis:** Wei Li, Lijun Wang

**Funding acquisition:** Yaokai Huang.

**Investigation:** Huaying Wang.

**Methodology:** Yaokai Huang, Wei Li

**Resources:** Yaokai Huang

**Software:** Lijun Wang

**Supervision:** Peng Tang.

**Validation:** Yaokai Huang, Peng Tang

**Visualization:** Huaying Wang

**Writing – original draft:** Wei Li, Huaying Wang.

**Writing – review & editing:** Peng Tang, Yaokai Huang.
